# Spatiotemporal analysis of severe fever with thrombocytopenia syndrome in Shandong Province, China, 2014–2018

**DOI:** 10.1186/s12889-022-14373-5

**Published:** 2022-11-01

**Authors:** Yao Wang, Bo Pang, Wei Ma, Zengqiang Kou, Hongling Wen

**Affiliations:** 1 Department of Epidemiology, School of Public Health, Cheeloo College of Medicine, Shandong University, Jinan, 250012 China; 2 Bacterial Infection Disease Control of Institute, Shandong Center for Disease Control and Prevention, Shandong Provincial Key Laboratory of Infectious Disease Prevention and Control, Jinan, 250014 China; 3 Department of Microbiological Laboratory Technology, School of Public Health, Cheeloo College of Medicine, Shandong University, Jinan, 250012 China

**Keywords:** Severe fever with thrombocytopenia syndrome, Spatial autocorrelation, Shandong Provicne, Cluster

## Abstract

**Background:**

Due to recent emergence, severe fever with thrombocytopenia syndrome (SFTS) is becoming one of the major public health problems in Shandong Province, China. The numbers of reported SFTS cases in general and the area with reported SFTS cases are both continuously increasing in recent years. However, spatiotemporal patterns and clusters of SFTS in Shandong Province have not been investigated yet.

**Methods:**

The surveillance data of SFTS in Shandong Province, China, during 2014–2018 were extracted from China Information System for Disease Control and Prevention (CISDCP). Geoda software was used to explore spatial autocorrelation analysis, and Satscan software was used to identify spatio-temporal clustering of cases. The results were presented in ArcMap.

**Results:**

The annual average incidence was 0.567/100,000 in Shandong Province during 2014–2018. Results showed that the distribution of SFTS was not random but clustered in space and time. A most likely cluster including 15 counties was observed in the northeastern region of Shandong Province from January 1, 2015 to December 31, 2015 (Relative risk = 5.13, Log likelihood ratio = 361.266, *P* < 0.001).

**Conclusions:**

The number of SFTS cases in Shandong Province increased overall. Geographic information system analysis coupled with spatial analysis illustrated regions with SFTS clusters. Our results provide a sound evidence base for future prevention and control programs of SFTS such as allocation of the health resources, surveillance in high-risk regions, health education, improvement of diagnosis and so on.

## Introduction

Severe fever with thrombocytopenia syndrome (SFTS) is an emerging tick-borne infectious disease caused by the SFTS virus (SFTSV) [1]. SFTS is one of the major public health problems for Eastern Asian countries, especially China, with a case fatality rate of 12–30% [2]. Due to climate change, increasing trend of urbanization, SFTS transmission has expanded in new geographic areas [3]. Unfortunately, there are no efficient therapeutics and vaccines against SFTSV currently. Therefore, understanding the dynamics of SFTS transmission seems imperative to reduce the public health burden.

*Haemaphysalis longicornis* (*H. longicornis*) is the predominant vector of SFTS in Shandong Province [2, 4]. The distribution of *H. longicornis* is closely related to the natural environment and has significant seasonal and regional characteristics [5]. Climate factors, such as humidity, temperature, and precipitation, affect the growth, development, activity, and survival rate of *H. longicornis* [5–7]. When environmental conditions are unsuitable or hosts are not available, ticks may enter a prolonged behavioral or development diapause [8]. Warmer temperatures have been suggested as the main driver of some tick geographic range changes [9]. Rising temperature has led to improved conditions for survival and reproduction of ticks and faster development leading to an acceleration of the tick lifecycle [5]. In addition, *H. longicornis* quest at variable heights in the vegetation, driven by factors such as temperature and relative humidity [10]. Due to variation in these factors, occurrence and spread of SFTS vary over space and time.

SFTS was first reported in China in 2009 and rapidly spread to other provinces in central, eastern, and northeastern regions [11]. Shandong Province is one of the world’s worst regions of SFTS [3]. Compared with other SFTS endemic areas of China, Shandong Province has distinctive geographical and climate environment. Shandong Province, lying in the transition between the humid subtropical and humid continental zones, has dry winter compared to other endemic regions with fully humid climate [12]. The unique geographical and climatic environments may significantly influence the distribution characteristics and transmission patterns of SFTS in Shandong Province. Hence, we assumed that SFTS cases in Shandong Province presented typical spatiotemporal pattern.

In recent years, geographic information systems and spatial statistics have been widely applied to describe the distribution characteristics and transmission patterns of diseases, which contributes to the timely surveillance and intervention of diseases, especially infectious diseases [13–16]. Many studies analyzing spatiotemporal patterns of diseases have used SaTScan and GeoDa public domain software. GeoDa software provides several ways to visualize and map distribution pattern of disease by correcting for spatial autocorrelation and spatial dependencies. SaTScan software provides a powerful tool to detect, delineate, and validate disease clusters.

However, few studies have been conducted in Shandong Province to explore the spatial epidemiological characteristics at the county level. Chang CY et al. (2022) analyzed the epidemiological characteristics and spatio-temporal clustering of SFTS in Jinan city of Shandong Province using SaTScan method [17]. Two studies just described the temporal, population and spatial distribution of SFTS in Qingdao city and Linyi city, respectively [18, 19]. However, these studies were confined to a specific region of Shandong Province with single method, which could not meet the requirement of prevention and control of SFTS in Shandong Province. Owing to economic conditions, climatic conditions, social factors, and geographical location, the spatiotemporal distribution features of tick-borne diseases vary in different regions [20, 21], and previous studies have demonstrated that the spatial distribution of SFTS can be quite heterogeneous within a country and even at subnational scales [16, 18, 19, 22]. Therefore, this study aims to explore the spatiotemporal pattern of SFTS based on surveillance data of Shandong Province from 2014 to 2018 to provide further basic data for scientific prevention and control against SFTS.

## Materials and methods

### Study area

**Fig. 1:**
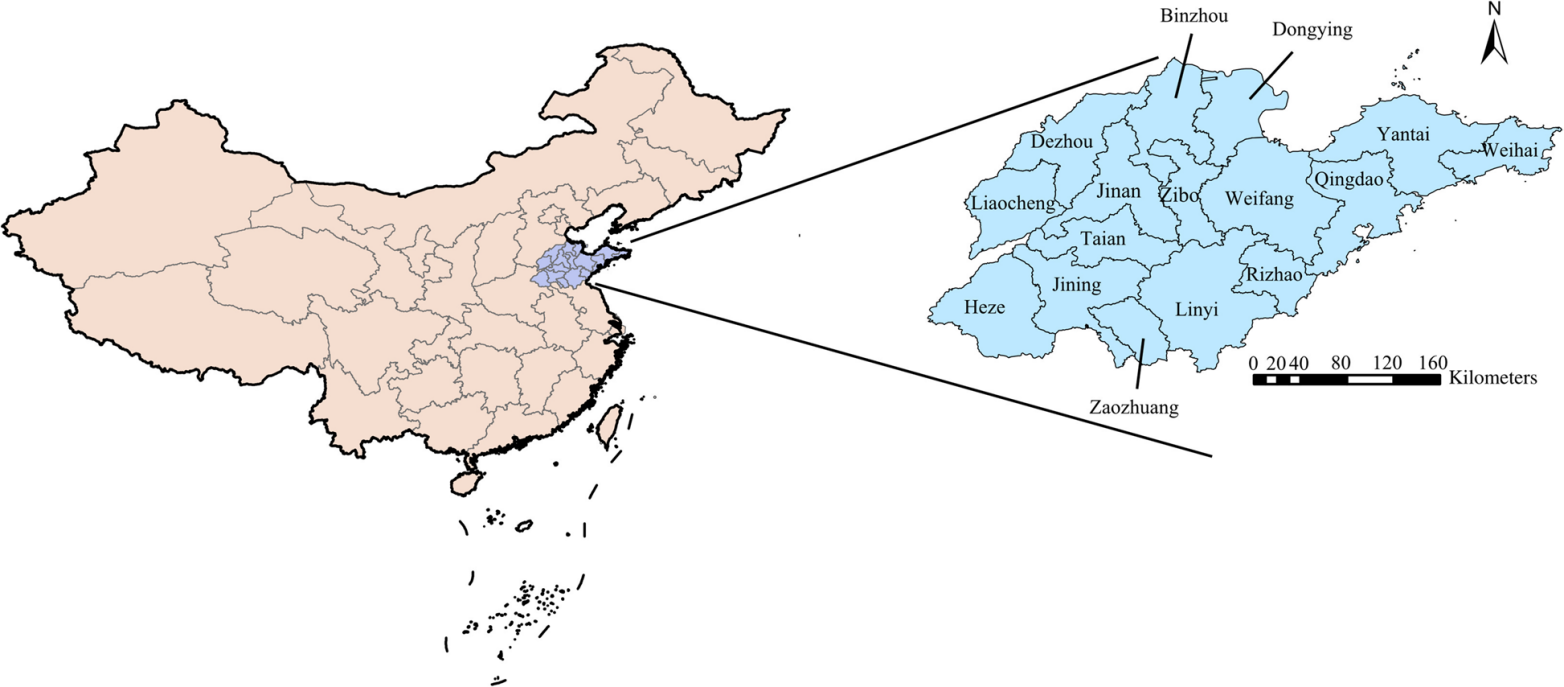
Location of the study area in China

Shandong Province has the highest burden of SFTS in China. Shandong Province is located in East China between latitudes 34°22.9′ N-38°24.01′ N and longitudes 114°47.5′ E-122°42.3′ E (Fig. 1), belonging to warm monsoon climate which has four distinctive seasons [12]. In the inland zone, annual precipitation ranges from about 20 inches (500 mm) in northwest Shandong to 40 inches (1,000 mm) as one approaches the mouth of the Huang He. Of the total annual precipitation, 70 to 80 percent falls in summer. Temperatures in the inland zone range from a mean January reading of 25 °F (− 4 °C) in the northern interior to a mean of 82 °F (28 °C) in July. There are two sub-provincial cities (Jinan and Qingdao) and fourteen prefecture-level cities, including Zibo, Zaozhuang, Dongying, Yantai, Weifang, Jining, Taian, Weihai, Rizhao, Binzhou, Dezhou, Liaocheng, Linyi and Heze. Shandong Province covers an area of 71,029.02 km^2^ and its population is 46.04 million in 2020.

### Data sources

By Chinese law, SFTS cases diagnosed in a hospital must be reported to China Information System for Disease Control and Prevention (CISDCP) within 24 h by the medical staff. The data on SFTS cases in Shandong Province from January 2014 to December 2018 were obtained from CISDCP. The patient diagnosis standard was in line with the China SFTS diagnosis and treatment guide published by Ministry of Health, China in 2010 [23]. The demographical data for each city of Shandong Province was downloaded from the Shandong Statistical Yearbook (http://tjj.shandong.gov.cn/col/col6279/index.html).

#### Data analysis

Excel 2016 and R 4.1.0 were used for analyzing and processing basic data. The SFTS incidence was defined as the number of cases divided by the total number of the population in each county. Geoda 1.20 was applied for spatial autocorrelation analysis and SaTScan 10.0.1 was used to identify the spatial patterns, temporal patterns, and clusters of SFTS in different counties and during different periods based on the Poisson probability model. A *P*-value less than 0.05 was considered significant.

#### Data visualization

We matched administrative codes of home addresses of SFTS cases to the map codes of the counties, combined with the map of cases, and the results were shown in ArcGIS 10.8.1. In addition, the results of spatial autocorrelation analysis and space–time scan analysis were also shown by ArcGIS software.

### Spatial autocorrelation tests

Spatial autocorrelation, generally including global and local spatial autocorrelation, is mainly used to evaluate the spatial correlation of geographical elements. In this paper, we used the global Moran’s *I* index [24] to explore the global correlation characteristics of reported SFTS cases in Shandong Province. The formula of Moran’s *I* index is as follows:

$$\mathrm{Global}\;\mathrm{Moran}'\mathrm s\;I=\frac{\sum_{i=1}^N\sum_{j=1}^N{\mathrm w}_{ij}\left({\mathrm x}_i-\overline{\mathrm x}\right)\left({\mathrm x}_j-\overline{\mathrm x}\right)}{S^2\sum_{i=1}^N\sum_{j=1}^N{\mathrm w}_{ij}}$$

where *N* is the total number of space units. *x*_*i*_ and *x*_*j*_ are attribute values, *W*_*ij*_ denotes the space weight matrix. In addition, the standard statistics *Z* was used to calculate the significance of the global Moran’s* I* index, and its formulas are as follows:

$$Z\;(\mathrm{Moran}'\mathrm s\;I)=\frac{Moran's\;I-E(Moran's\;I)}{\sqrt{VAR}\left(Moran's\;I\right)},\;\mathrm{where}\;E\;(\mathrm{Moran}'\mathrm s\;I)\;=-\frac1{N-1}$$

The value of global Moran’s* I* is [-1,1]. There will be global autocorrelation between cities if the index is significantly greater than 0. The larger this index is, the stronger the agglomeration effect of SFTS cases will be. There is no spatial autocorrelation When Moran’s* I* is equal to 0. The local Moran’s* I* index was used to further analyze the local spatial correlation of reported SFTS cases, and its formula is as follows:

$$\mathrm{Local}\;Moran's\;I\;=\;\frac{Z_i}{S^2}{\textstyle\sum_{j\neq i}^N}W_{ij}Z_j$$

where ${Z}_{i}={y}_{i}-\overline{y }$, ${Z}_{j}={y}_{j}-\overline{y }$, ${S}^{2}=\frac{1}{N}\sum {({y}_{i}-\stackrel{-}{y)}}^{2}$, *y* represents reported SFTS cases, $\overline{y }$ and *S*^2^ represents its mean and variance, respectively. Four categories of local spatial relations are obtained according to the relationship between local spatial unit and its adjacent spatial units, namely positive spatial correlation (High-High and Low-Low modes) and negative spatial relationship (High-Low and Low–High modes).

### Space–time scan analysis

SatScan software version 10.0.1 developed by Kuldlorff was used to detect and evaluate SFTS clusters. SaTScan is a free software for finding regions in time, space, or time–space that have excess risk, based on scan statistics, and uses Monte Carlo hypothesis testing to generate *P*-values for these regions [25]. SaTScan scans gradually across time and/or space to identify possible clusters by comparing the number of observed incidences and expected incidence (assuming random distribution) inside the window at each location. Scanning window is a time interval for purely temporal scan, a circle or ellipse in spatial scan and a cylinder in space–time scan where base of a cylinder represents space dimension and height represents the temporal dimension. The null hypothesis is that the risk of SFTS incidence is equal throughout the study area while the alternative hypothesis is that the risk of SFTS is different inside and outside of at least one circle or cylinder. The cluster with the maximum log likelihood ratio (LLR)is taken as the most likely cluster, i.e. the cluster least likely to be due to chance. The LLR in Possion distribution is computed as:

$$LLR=\;(\frac C{E\left(c\right)}c\cdot\left(\frac{C-c}{C-E\left(c\right)}\right)c\cdot I()$$

where:

*LLR* = Log Likelihood Ratio

*C* = total number of cases

*c* = observed number of cases within the window

*E(c)* = covariate adjusted expected number of cases within the window under the null hypothesis

*I()* = indicator function

In this study, we set county as the smallest scanning unit using the three-dimensional dynamic changing cylinder scan window to analyze data. Purely space scan statistics impose a circular window on the map. The spatiotemporal scan analysis sets the radius of the circular window as the geographic position and the size of the region, and the height corresponds to the scanning time.

The maximum cluster size was set to 50% of the population at risk for spatial scan; to account for differences in population density and a non-overlapping secondary cluster was set to be reported. In temporal scan analysis, a value of one year was chosen for the maximum temporal window size to capture seasonality in SFTS incidence. Monte Carlo was used to calculate the P-value of the test statistics, and the simulation times were set to 999. The cluster group with maximum LLR as the main cluster, and the others are secondary clusters in order.

## Results

### Descriptive results

**Fig. 2:**
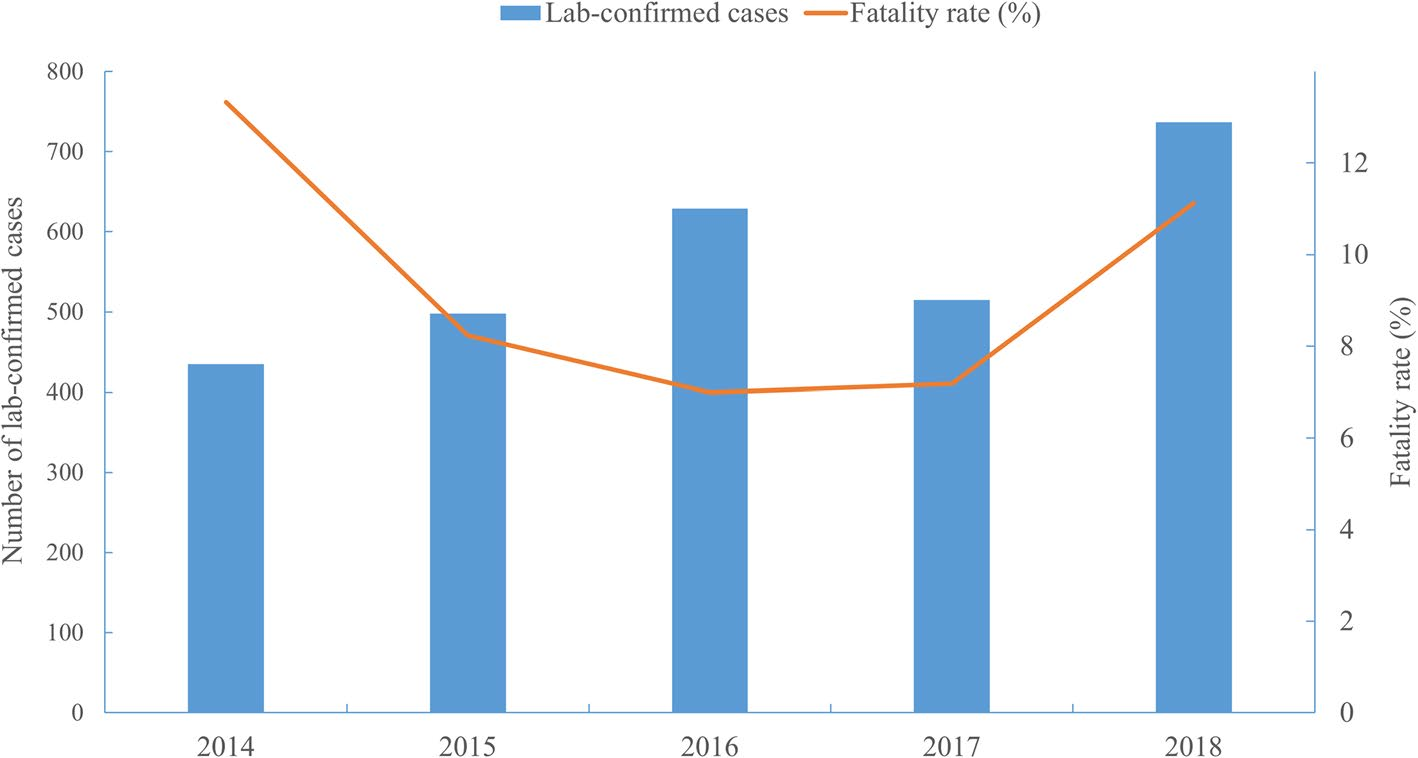
The number of SFTS cases and the fatality rate in Shandong Province from 2014 to 2018

**Fig. 3:**
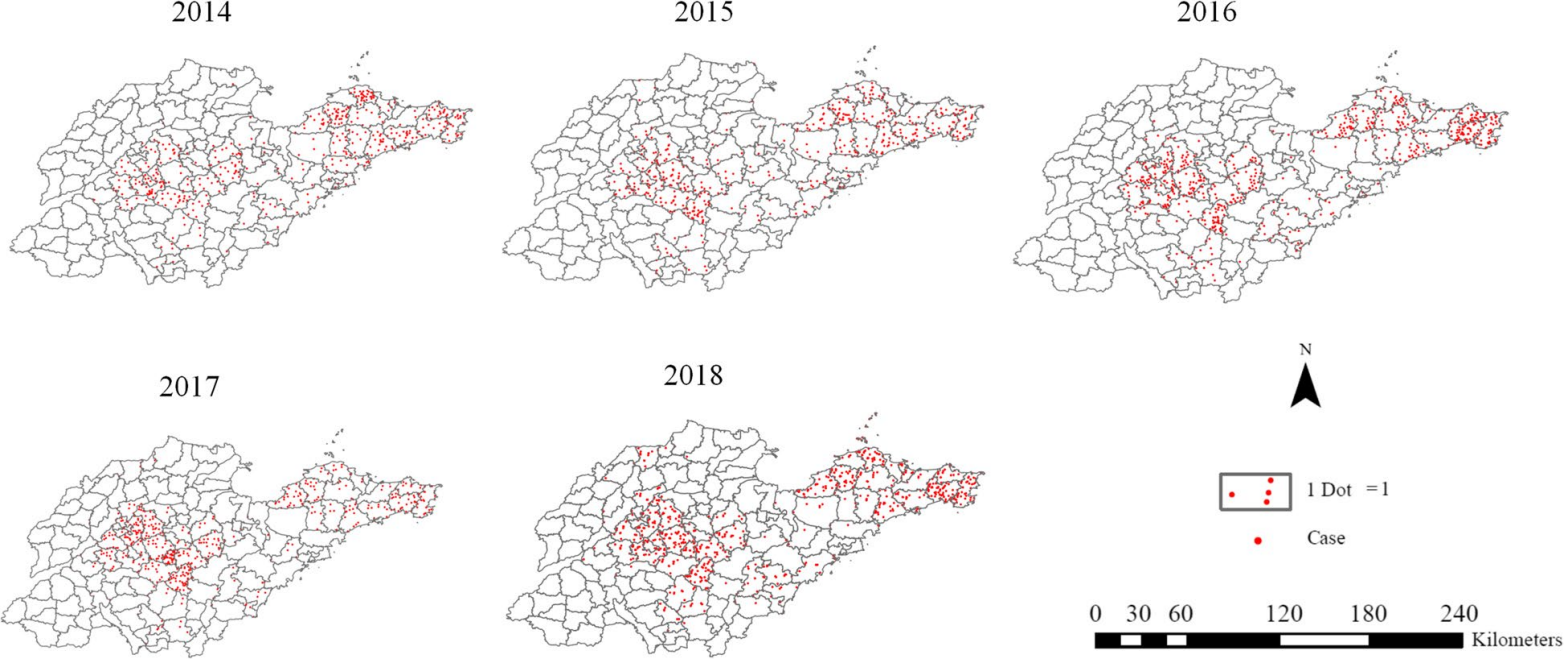
The spatial distribution of SFTS cases in Shandong Province from 2014 to 2018. 1 dot represents one reported case

**Fig. 4:**
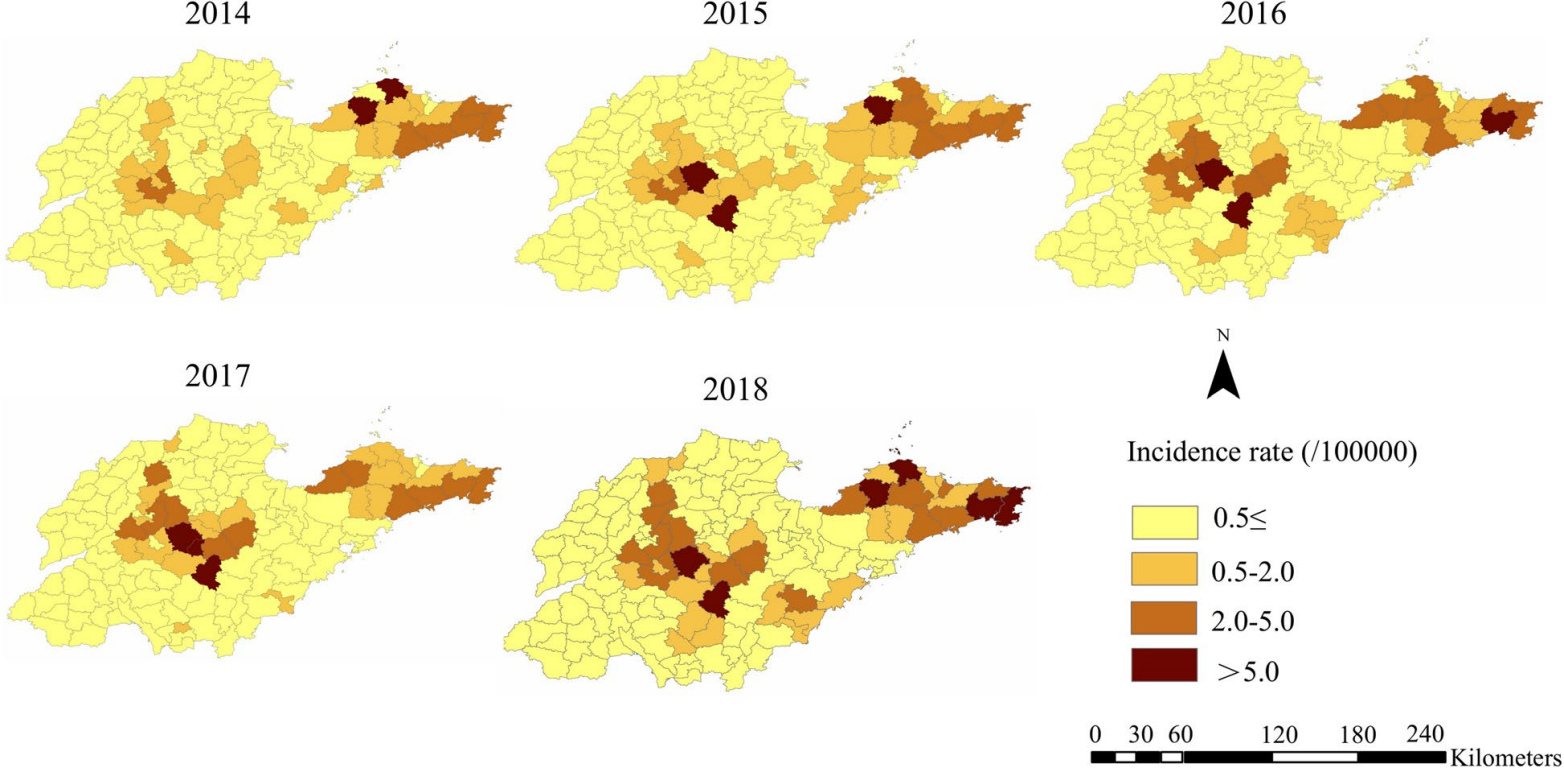
The incidence rate of SFTS in different regions of Shandong Province from 2014 to 2018

**Fig. 5:**
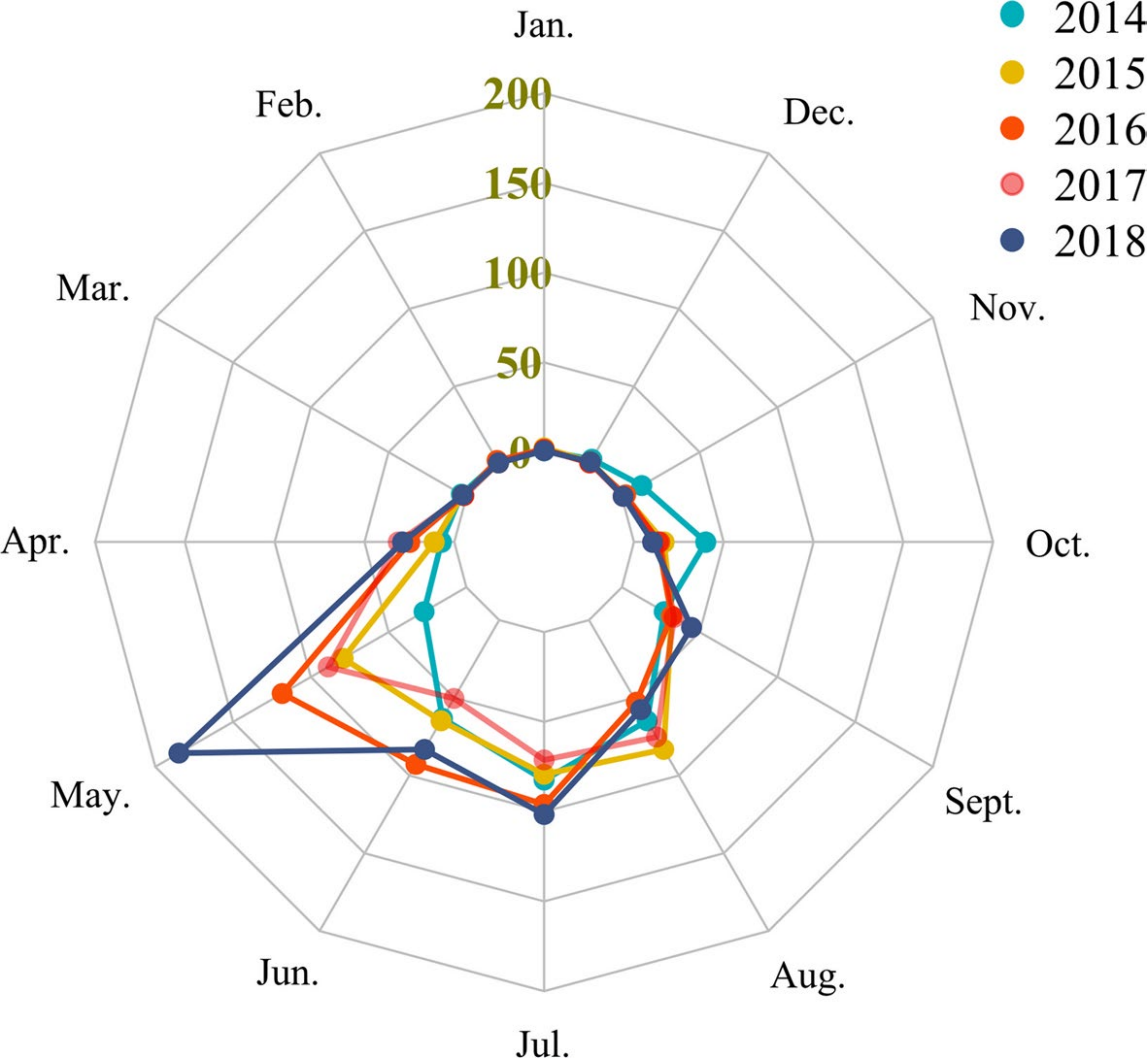
The temporal distribution of SFTS cases in different cities of Shandong Province

A total of 2814 cases were reported in Shandong Province from 2014 to 2018, of which fatal cases accounted for 8.99% (253/2814). The annual fatality rate and the number of confirmed cases showed an opposite changing trend (Fig. 2). Yantai (666 cases), Jinan (574 cases), Weihai (451 cases), Taian (302 cases) and Linyi (250 cases) took the top five spots (Table 1). The incidence interval ranged from 0.463/100,000 (2014) to 0.734/100,000 (2018), with an annual average incidence of 0.567/100,000. SFTS cases were concentrated in central and eastern Shandong Province with significant spread trend (Fig. 3). The spatial distribution of annual SFTS incidence revealed that regions with higher incidence than average five-year incidence are similar. Laiwu district of Jinan city, Mengyin district of Linyi city, Penglai city and Zhaoyuan city of Yantai city, Wendeng and Rongcheng city of Weihai city were the most affected areas (Fig. 4). The time distribution of reported cases showed obvious seasonal characteristics that were characterized by rising rapidly trend in April and peak period from May to October (Fig. 5).

**Table 1:** Number of SFTS cases during 2014–2018 in different cities of Shandong Province

	2014		2015		2016		2017		2018	
	Report cases	Fatal cases	Report cases	Fatal cases	Report cases	Fatal cases	Report cases	Fatal cases	Report cases	Fatal cases
Jinan	36	7	74	8	117	9	130	12	151	15
Qingdao	42	4	53	3	21	4	15	2	22	1
Zibo	14	1	16	3	23	0	29	1	34	2
Zaozhuang	6	0	9	0	7	0	7	0	8	0
Dongying	1	0	4	0	0	0	0	0	0	0
Yantai	125	25	130	15	143	16	90	15	178	31
Weifang	44	0	25	1	49	1	41	0	37	0
Jining	0	0	1	0	2	0	4	0	0	0
Taian	69	13	60	3	60	8	51	2	62	0
Weihai	70	6	64	5	123	5	65	3	129	19
Rizhao	12	2	7	0	24	1	14	0	30	0
Linyi	15	0	52	3	58	0	59	1	66	0
Dezhou	0	0	3	0	0	0	7	1	18	5
Liaocheng	0	0	0	0	0	0	0	0	0	0
Binzhou	1	0	0	0	2	0	3	0	2	0
Heze	0	0	0	0	0	0	0	0	0	0

### Spatial autocorrelation analysis

**Fig. 6:**
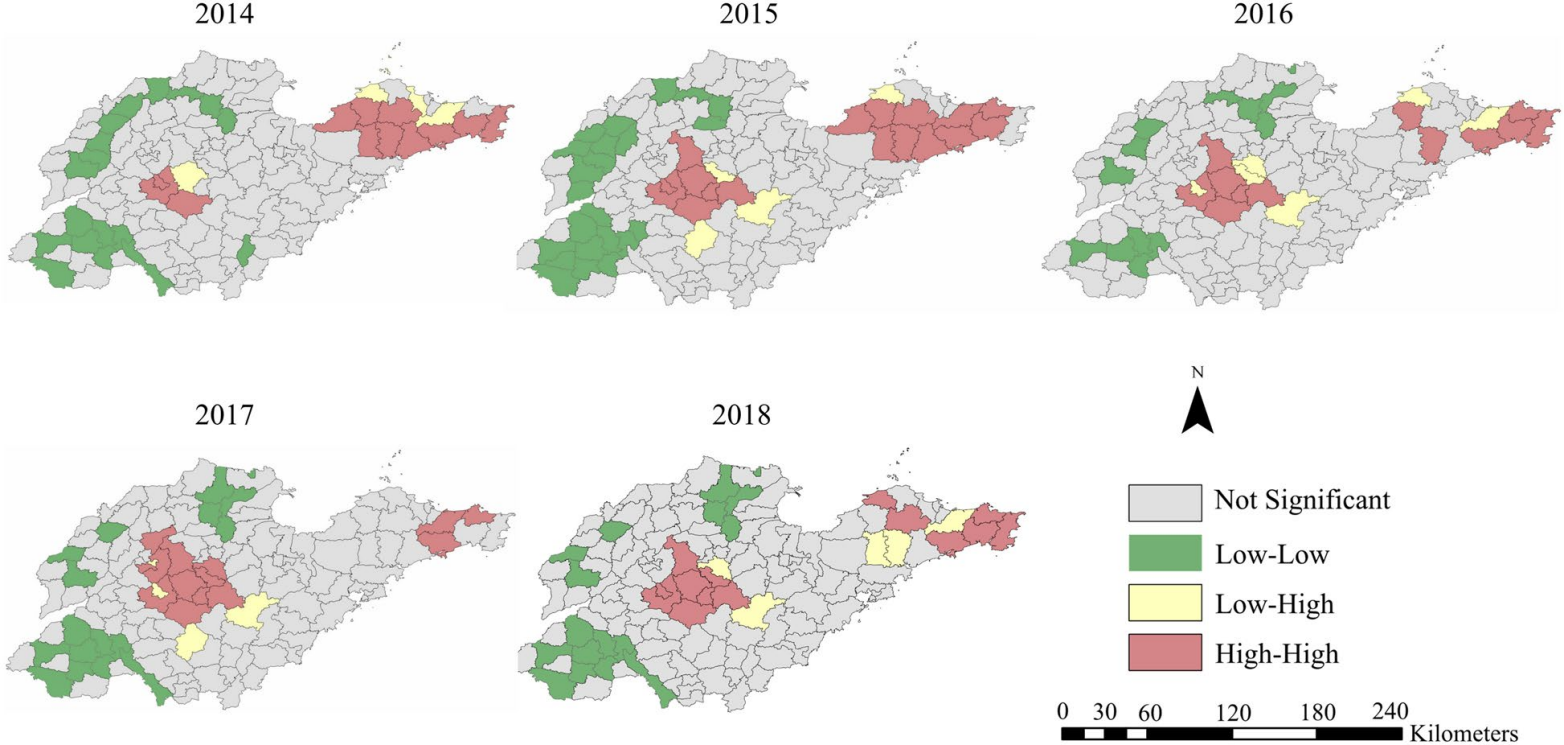
The spatial–temporal of SFTS agglomeration areas in Shandong Province from 2014 to 2018

The global spatial autocorrelation results were depicted in 2014–2018 (Table 2). The Moran’s index of SFTS incidence in Shandong Province from 2014 to 2018 was all positive. The Moran’s index passed the significance test of 1%. These results can effectively show that the SFTS incidence in Shandong Province had a spatial distribution characteristics of aggregation and SFTS cases of sixteen cities in Shandong Province had a significant positive spatial correlation. The value of Moran’s index in 2018 was the largest, indicating that the agglomeration of SFTS incidence was the most obvious. Local spatial autocorrelation analysis for SFTS significant cluster map showed the presence of three kinds of clusters: high-high, low-low, and low–high clusters. High-high clusters were mainly distributed in Yantai, Weihai, Jinan, and Taian, whereas low-low clusters were mainly distributed in western, northern, and southwestern regions of Shandong Province (Fig. 6). Compared with the high-high clusters located in the central regions, the high-high clusters located in the eastern regions varied widely from 2014 to 2018. Significantly, Wendeng district of Qingdao city consistently belonged to the high-high clusters. However, local spatial autocorrelation analysis also identified some abnormal areas located in the central and northeastern regions of Shandong Province. Yishui county was detected as low–high cluster region from 2015 to 2018, and Longkou city belonged to the low–high cluster region from 2014 to 2016. More specifically, high-high agglomeration areas included twelve counties/districts of four cities in 2014, seventeen counties/districts of six cities in 2015, twelve counties/districts of five cities in 2016, fourteen counties/districts of five cities in 2017, and fourteen counties of five cities in 2018 (Fig. 6, Table 3).

**Table 2:** Moran’s index distribution of SFTS incidence in Shandong Province during 2014–2018

Year	I	E[I]	mean	Z-value	*P*-value
2014	0.399	-0.0075	-0.0056	7.3993	< 0.001
2015	0.454	-0.0074	-0.0085	8.6654	< 0.001
2016	0.426	-0.0074	-0.0091	8.0843	< 0.001
2017	0.356	-0.0074	-0.0092	7.1144	< 0.001
2018	0.464	-0.0074	-0.0103	8.9975	< 0.001

**Table 3:** Distribution of high-high agglomeration areas of SFTS incidence in Shandong Province from 2014 to 2018

Year	Number of counties/districts	City	County/District
2014	12	Qingdao, Yantai, Taian, Weihai	Laixi, Laiyang, Lizhou, Zhaoyuan, Qixia, Haiyang, Taishan,Daiyue, Xintai, Wendeng, Rongcheng, Rushan
2015	17	Jinan, Qingdao, Zibo, Yantai, Taian, Weihai	Zhangqiu, Laiwu, Gangcheng, Laixi, Yiyuan, Muiping, Laiyang, Laizhou, Zhaoyuan, Qixia, Haiyang, Taishan, Daiyue, Xintai, Huancui, Wendeng, Rushan
2016	12	Jinan, Zibo, Yantai, Taian, Weihai	Zhangqiu, Laiwu, Gangcheng, Yiyuan, Laiyang, Zhaoyuan, Daiyue, Xintai, Huancui, Wendeng, Rongcheng, Rushan
2017	14	Jinan, Zibo, Yantai, Taian, Weihai	Shizhong, Licheng, Zhangqiu, Jiyang, Laiwu, Gangcheng, Zichuan, Boshan, Yiyuan, Muping, Daiyue, Xintai, Huancui, Rushan
2018	14	Jinan, Zibo, Yantai, Taian, Weihai	Zhangqiu, Laiwu, Gangcheng, Boshan, Yiyuan, Longkou, Qixia, Taishan, Daiyue, Xintai, Huancui, Wendeng, Rongcheng, Rushan

### Spatiotemporal clustering analysis

**Fig. 7:**
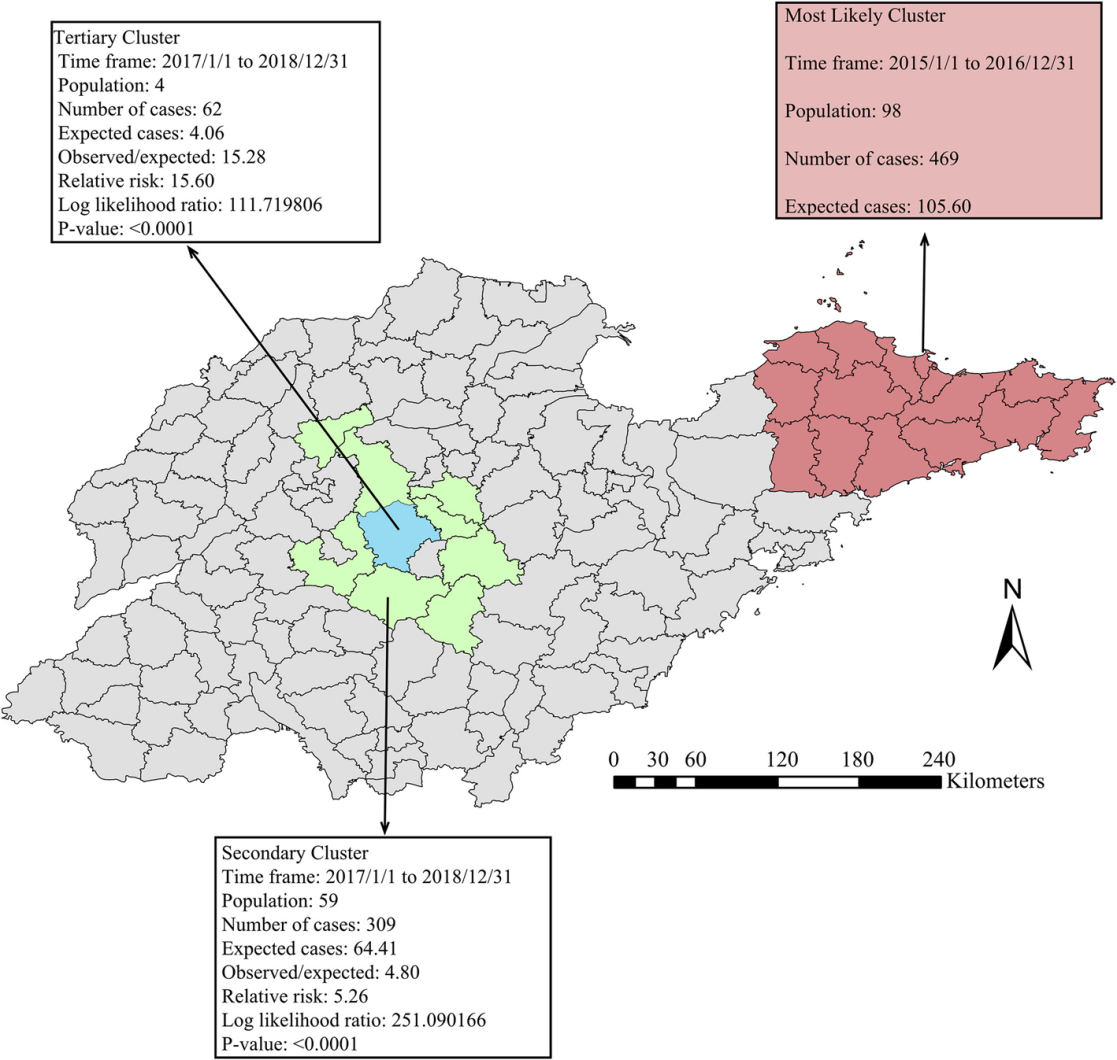
Yearly detected significant spatial–temporal clusters for areas with high rates of SFTS in Shandong Province from 2014 to 2018

Spatiotemporal scans using SaTScan were used to analyze SFTS occurrences during 2014–2018. The results showed that the incidence of SFTS was spatiotemporally clustered. One probably primary cluster, one secondary cluster, and one tertiary cluster are shown in Fig. 7.

The most likely cluster was mainly distributed in the eastern Shandong Province and covered 17 counties (Zhifu district, Laishan district, Fushan district, Muping district, Panglai city, Qixia city, Wendeng district, Rushan city, Huancui district, Longkou city, Haiyang city, Changdao county, Zhaoyuan city, Laiyang city, Rongcheng city, Laixi city and Laizhou city), with a relative risk (RR) of 5.13 and LRR of 361.266 (*P* < 0.001). The cluster time was from January 1, 2015, to December 31, 2015. The secondary and tertiary clusters were both distributed in the central areas of Shandong Province. The secondary cluster covered eight counties (Jiyang district, Xintai city, Zhangqiu district, Yiyuan county, Boshan district, Mengyin county, Zichuan district and Daiyue district) located in four cities (RR = 5.26, LRR = 251.090, *P* < 0.001), and the tertiary cluster entirely surrounded by the secondary cluster covered one county (Laiwu district) (RR = 15.60, LRR = 1111.720, *P* < 0.001).

## Discussion

Shandong Province is one of the key epidemic areas where SFTS was first discovered, and monitoring work is carried out in accordance with the requirements of category B infectious disease under the Law of the People’s Republic of China on prevention and control of infectious diseases. In this study, exploratory data analysis and spatiotemporal cluster analysis of SFTS were conducted at the county level in Shandong Province, China. We mapped SFTS in terms of reported number and crude incidence. In addition, we further evaluated spatiotemporal distribution patterns and explored significant spatial, temporal, and spatiotemporal clusters.

Both the reported number and incidence of SFTS in Shandong Province increased steadily over the five-year study period. We also saw an expansion of epidemic zone around Shandong Province, reflecting the increasing spread of the disease. Miao et al. [22] found that this increasing spread could be a result of intense exposure to circulating SFTSV through field activity, but increased surveillance, vigilance, and awareness of the disease might also be responsible. Similar to the results of previous studies [16, 18–20, 26, 27], most SFTS cases occurred from April to October. The seasonal distribution of SFTS cases might relate to tick dynamics [28–30]. Ticks were reported as the carriers and vector host of SFTSV, of which *H. longicornis* were the predominant population carrying SFTSV [11, 31]. *H. longicornis*, the dominant tick in Shandong Province, accounted for about 75% of all the collected ticks in Shandong Province [32]. *H. longicornis* are active in early March on vegetation, and a number of *H. longicornis* achieve peak in September, with nymphs active in early April and reaching peak in May and adults active from March to September and reaching peak in July [6, 33]. According to the spatial distribution analysis, the distribution pattern of incidence of SFTS showed significant spatial heterogeneity. The high incidence regions of SFTS were mainly located in eastern and central regions. Annual spatial monitoring in endemic regions can significantly contribute to the identification of SFTS foci and increasing intensity and degree of targeted control measures. In addition, significant annual variations in the incidence of SFTS among regions highlight the presence of pseudosilent areas and a lack of effective surveillance in low-epidemic areas [17]. These abrupt changes in the epidemiological scenario of SFTS may reinforce the problem of local underdiagnoses and, in part, justify the results found in this study [17].

Spatial autocorrelation analysis demonstrated statistically significant, suggesting a non-random spatial clustering pattern of SFTS in Shandong Province. The local spatial autocorrelation analysis showed that the hotspots were located in the eastern and central regions, which was essentially consistent with the high incidence of SFTS in Shandong Province. Previous study demonstrated that SFTS incidence in Shandong Province was associated with local agricultural activities [20]. Farmers who live in eastern and central Shandong Province are more engaged in raising goats, which were the major animal host of *H. longicornis*, the dominant tick in Shandong Province and the vector of SFTSV [32, 34]. A study found that eastern and central regions of Shandong Province belonged to the high-suitable habitats of *H. longicornis* [21, 35]. In summary, the people living in these areas have more opportunities to exposure to SFTSV. However, some areas with similar geographical and climatic environments to high-high cluster regions showed lower SFTS incidence. Further investigations are necessary to give more accurate answers about these anomalous regions, such as the virus-carrying rate of ticks, and the species and density of hosts.

The spatiotemporal scan analysis detected three significant clusters of SFTS incidence, which were mainly located in the central and northeastern of Shandong. Although SFTS disease is spreading rapidly to new areas, it is highly localized in particular locations and times. Types of farming, suitable climate, and natural environment may contribute to the high SFTS cluster [20, 21, 36]. High-prevalence areas of SFTS in Shandong Province are mostly located in mountainous and hilly regions where local people graze animals and the density of ticks is high [37]. In addition, free-range domestic animals are more susceptible to SFTSV due to the greater chances of exposure to tick bites [38]. A meta-analysis showed that the seroprevalence of SFTSV in free-range animals was significantly higher than that in animals that had a confined range [39]. As intermediate hosts, domestic animals, especially the goats that play an important role in agricultural activities in mountainous and hilly areas, were found to contribute significantly to the spread of SFTSV [40]. However, more studies to understand SFTSV transmission in nature are still necessary because of limited knowledge of animal-to-animal transmission and animal-to-human transmission. Moreover, with the enhancement of ecological protection in recent years, vegetation coverage and biodiversity have increased significantly, which is beneficial to the survival and reproduction of ticks, highlighting the importance of health education for high-risk groups [41].

Compared to previous studies, this study has several notable strengths. First, acknowledging the inherent limitations of all spatial analysis techniques, we used complementary methods to achieve greater accuracy. Therefore, this study could be an excellent example to facilitate such studies at higher temporal and spatial scales in the future. Second, based on the surveillance data of entire regions of Shandong Province, our results could be valuable to the public health authority to design and execute an intervention program on SFTS in Shandong Province. However, there are also several limitations with this study. First, national SFTS surveillance relies on passive reporting and clinician awareness, which can result in underreporting. Such as those who did not come to health facilities for treatment, and poor cooperation of private health institutions in the government reporting system. Fortunately, this situation has improved remarkably in recent years. Second, this study did not analyze possible environmental risk factors (SFTSV-carrying rate of ticks and meteorological factors) which contribute to the spatial cluster of SFTS and therefore we could not pinpoint such risk factors. However, we were not able to obtain data on these factors in this study, and additional researches are warranted to elucidate the factors associated with SFTS incidence. Third, mapping and analysis on coarsely aggregated data, month and district, may have missed daily or weekly local SFTS clusters. If we had daily or weekly dengue cases at lower spatial unit, we could detect outbreak dynamics and movements of hotspots.

## Conclusions

This study assessed and mapped the spatiotemporal patterns of SFTS in Shandong Province using geographic information systems and spatial statistics methods. Our study confirmed that (1) the annual number of SFTS cases and affected counties showed an increasing tendency in Shandong Province from 2014 to 2018, (2) spatial distribution of SFTS in Shandong Province was not random, with high-high clusters locating in the central and eastern regions. This study clearly demonstrates the importance of geospatial techniques in mapping and spatiotemporal assessment of infectious diseases. The method adopted here can be used for other diseases and higher spatiotemporal scales. Moreover, our study provides spatial information for the exploration of influencing factors which were associated with SFTS incidence. Our results suggest that more manpower and material resources should be allocated to prevent and control the high incidence areas of SFTS more effectively, especially in Jinan, Taian, Weihai and Yantai. Early identification and prevention of high-incidence areas will improve the efficiency of SFTS control and management in Shandong Province. Meanwhile, we recommend active health education be placed in regions located in the above regions. The improvement of diagnosis in these regions is also necessary.

## Data Availability

The datasets used and/or analysed during the current study are available from the corresponding author on reasonable request.

## Abbreviations

SFTS: Severe fever with thrombocytopenia syndrome
SFTSV: Severe fever with thrombocytopenia syndrome virus
CISDCP: China Information System for Disease Control and Prevention
RR: Relative risk
LLR: Logarithmic likelihood ratio
SaTScan: Spatiotemporal scan
